# Effect of Interfacial Schemes on the Optical and Structural Properties of InAs/GaSb Type-II Superlattices

**DOI:** 10.1021/acsami.2c19292

**Published:** 2023-02-01

**Authors:** Dhafer Alshahrani, Manoj Kesaria, Juan J. Jiménez, Dominic Kwan, Vibha Srivastava, Marie Delmas, Francisco M. Morales, Baolai Liang, Diana Huffaker

**Affiliations:** † School of Physics and Astronomy, Cardiff University, The Parade, CardiffCF24 3AA, U.K.; ‡ California NanoSystems Institute, University of California, Los Angeles, California90095, United States; § Department of Materials Science and Metallurgical Engineering and Inorganic Chemistry, Faculty of Sciences, University of Cádiz, Puerto Real11510, Cádiz, Spain; ∥ IMEYMAT: Institute of Research on Electron Microscopy and Materials, University of Cádiz, Puerto Real11510, Cádiz, Spain

**Keywords:** InAs/GaSb, type-II superlattice, InSb-like interface, photoluminescence, transmission electron microscopy, band structure simulation, atomic intermixing, segregation

## Abstract

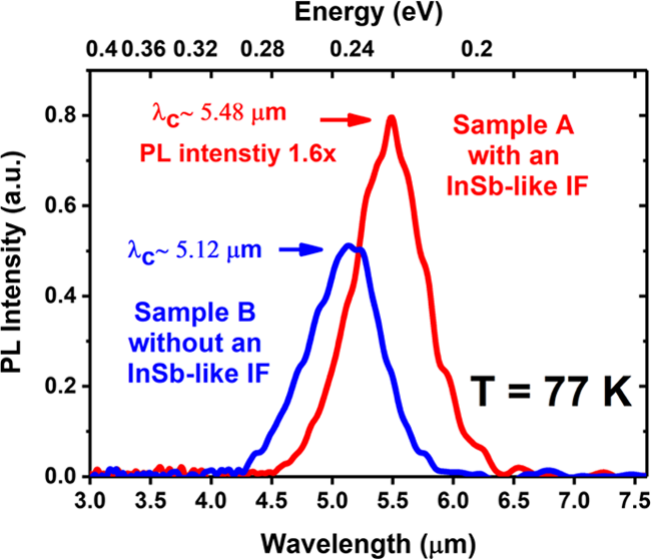

Incorporating an intentional strain compensating InSb interface (IF) layer in InAs/GaSb type-II superlattices (T2SLs) enhances device performance. But there is a lack of studies that correlate this approach’s optical and structural quality, so the mechanisms by which this improvement is achieved remain unclear. One critical issue in increasing the performance of InAs/GaSb T2SLs arises from the lattice mismatch between InAs and GaSb, leading to interfacial strain in the structure. Not only that but also, since each side of the InAs/GaSb heterosystem does not have common atoms, there is a possibility of atomic intermixing at the IFs. To address such issues, an intentional InSb interfacial layer is commonly introduced at the InAs-on-GaSb and GaSb-on-InAs IFs to compensate for the strain and the chemical mismatches. In this report, we investigate InAs/GaSb T2SLs with (Sample A) and without (Sample B) InSb IF layers emitting in the mid-wavelength infrared (MWIR) through photoluminescence (PL) and band structure simulations. The PL studies indicate that the maximum PL intensity of Sample A is 1.6 times stronger than that of Sample B. This could be attributed to the effect of migration-enhanced epitaxy (MEE) growth mode. Band structure simulations understand the impact of atomic intermixing and segregation at T2SL IFs on the bandgap energy and PL intensity. It is observed that atomic intermixing at the IFs changes the bandgap energy and significantly affects the wave function overlap and the optical property of the samples. Transmission electron microscopy (TEM) measurements reveal that the T2SL IFs in Sample A are very rough compared to sharp IFs in Sample B, indicating a high possibility of atomic intermixing and segregation. Based on these results, it is believed that high-quality heterostructure could be achieved by controlling the IFs to enhance its structural and compositional homogeneities and the optical properties of the T2SLs.

## 1 Introduction

Mid-wavelength infrared (MWIR) emitters and detectors find applications in spectroscopy, gas sensing, medical diagnostics, space and astronomy, defense, and night vision. In particular, environmental pollution caused by toxic gases such as carbon dioxide (CO_2_), carbon monoxide (CO), methane (CH_4_), nitrogen dioxide (NO_2_), and nitric oxide (NO) is one of the significant global challenges for humanity.^1^ These gases have strong absorption signatures in the MWIR spectral band between 3 and 6 μm, which drives demand for sensitive and powerful MWIR technologies.

The materials for current state-of-the-art technologies in the MWIR regime are mercury cadmium telluride (MCT), InSb, and InAsSb. MCT has several drawbacks, including Hg and Cd toxicity,^2^ poor uniformity,^3^ high costs of growth and fabrication,^3^ and low producibility yield.^3^ InSb can only operate at low temperatures, typically between 80 and 100 K,^4^ and InAsSb suffers from the lack of a suitable lattice-matched substrate and a high tunneling current. Due to these constraints, there is a need for an alternative competing material system. Superlattice (SL) is a periodic heterostructure of two or more alternating layers whose bandgap can be engineered by changing the thickness of the constituent layers. Type-II superlattice (T2SL) is emerging as a popular material for photodetectors (PDs),^5−7^ photodiodes,^8−10^ avalanche photodetectors (APDs),^11,12^ light-emitting diodes (LEDs),^13,14^ lasers,^15−17^ and phototransistors.^18,19^ Due to its advantages, such as suppressed Auger recombination,^20,21^ reduced tunneling current,^22^ and the flexibility of incorporating unipolar barriers,^23^ T2SL-based devices are theoretically expected to achieve higher performance levels than MCT detectors.^24,25^

**Figure 1:**
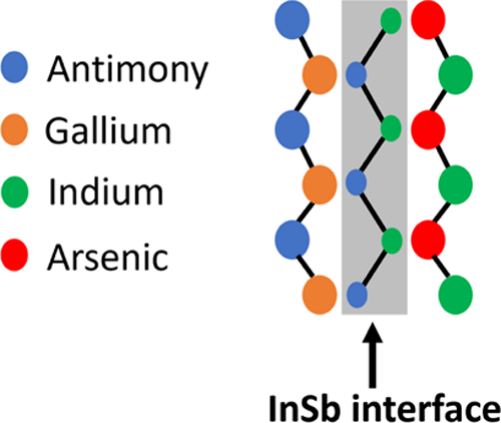
Schematic of an InSb-like IF in InAs/GaSb T2SL.

However, one of the obstacles to increasing the performance of InAs/GaSb T2SLs devices arises from the −0.6% lattice mismatch between InAs and GaSb, resulting in an interfacial strain that limits the material quality and device thickness. Therefore, the nature and sharpness of the interfacial elements have been an active area of study. The heterostructure of InAs/GaSb does not have common cations or anions. Still, it can have various interface (IF) schemes, namely, InSb-like or GaAs-like, or a combination of In, Ga, As, and Sb leading to ternary or quaternary interfacial intermixing,^26,27^ In and Sb segregation,^28,29^ and interface-diffusion of atoms.^30^ At the IFs, there is a possibility for forming either GaAs-like or InSb-like IF since the group-V (As and Sb) and group-III (In and Ga) atoms have different atomic kinetic energy at the growth temperature. When the GaAs-like IF is formed, the average lattice constant of the SL decreases even further, thereby increasing the tensile strain in the SL. Unlike the formation of the InSb-like IFs, the average lattice constant of the SL increases slightly, which could partially compensate for the strain in the SL. Promising device performance reported in the literature with IFs such as GaAs-like,^31−34^ InSb-like,^35−41^ and ternary/quaternary.^41,42^ Out of these, the InSb-like IF is the most implemented scheme due to its strain compensation ability,^43^ which arises from the +6.3% lattice mismatch between InSb and GaSb, acting against the −0.6% lattice mismatch between InAs and GaSb. Theoretically, strain compensation in InAs/GaSb T2SL can be achieved by choosing the InSb IF thickness around 10% of the InAs thickness. The schematic shown in Figure 1 depicts an InSb-like IF formed by sandwiching the InSb layer between InAs and GaSb layers. The terminology “InSb-like IF” in InAs/GaSb T2SLs is described in ref (44). The InSb-like IF also preserves the type-II band alignment in InAs/GaSb SL, which is advantageous for its structural and optical quality. The InSb-like IF is obtained using a migration-enhanced epitaxy (MEE) or conventional molecular beam epitaxy (MBE). However, T2SLs with InSb-like IF have shown superior optical quality despite the inferior structural quality compared to other schemes.^45,46^ To understand why this is the case, there is a need for a comparative optical and structural study of InAs/GaSb T2SLs with and without InSb IFs.

Previously, we reported growth details of MWIR T2SLs with two different IF schemes using two different shutter sequences.^46^ One sample was grown by the MEE technique with an intentional InSb IF layer, and the other with the Sb-for-As exchange technique but without an intentional InSb IF layer. Here, we report a detailed optical and structural study of 7 MLs InAs/4 MLs GaSb T2SLs with (Sample A) and without (Sample B) InSb-like IF layers, where MLs are monolayers. The IF effect on the optical and structural quality of InAs/GaSb T2SL is probed in this work using photoluminescence (PL) measurements, band heterostructure simulations, and transmission electron microscopy (TEM) measurements.

## 2 Methods

### 2.1 Growth of Samples

The samples investigated in this study were grown in a Veeco Gen 930 by MBE reactor. Sample A was grown with an intentional InSb IF layer at both SL IFs to compensate for the strain from the GaSb layer on the InAs layer using the MEE growth method. In contrast, Sample B was grown with no intentional InSb IF layer. However, Sb flux was only soaked for 6 s after the growth of the InAs layer to potentially promote an InSb-like IF at the GaSb-on-InAs SL IF, using an Sb-for-As exchange growth technique. Both samples were grown on GaSb substrates and buffer layers with a thickness of 50 nm. The active region comprises 100 periods of 7 MLs InAs/4 MLs GaSb T2SL, capped with a 1.2 nm undoped GaSb layer.

### 2.2 Photoluminescence

For the optical characterization measurements, the samples were mounted onto a liquid nitrogen (LN_2_) cryostat equipped with CaF_2_ windows to perform temperature-dependent PL measurements. A laser diode with a wavelength of 785 nm was used as an excitation power source, and the laser power was fixed at 50 mW. A pulse generator was used to modulate the excitation source at a frequency of 20 kHz, and a lock-in amplifier was then used to subtract the background signal. To acquire the PL signal from the samples, a Nicolet iS50R Fourier transform infrared (FTIR) spectrometer was utilized, and a cooled MCT detector then detected the acquired PL signal.

### 2.3 Band Heterostructure Simulation

The 8-band *k*·*p* envelope function method is implemented in Nextnano3 software.^47^ The IF matrix, formulated by Klipstein et al.,^48^ was implemented within the software framework. For a no-atom-in-common interfacial system such as InAs/GaSb, the matrix is defined as
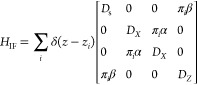
1where *i* is the index of the IF at the position *z*_*i*_ and π_*i*_, which takes a value of −1 or 1 at the GaSb-on-InAs and InAs-on-GaSb IFs. In line with Livneh et al.,^49^ the IF parameters *α* and *β* were fixed at 0.2 eV·Å. The diagonal IF parameters *D*_s_, *D*_*x*_, and *D*_*z*_, were chosen for best agreement with PL results from previously reported SL structures.^50^ The parameters used for InAs, GaSb, and InSb can be found elsewhere.^51^ The bowing parameter for the conduction and valence band energies for InAsSb were +0.65 and −0.98 eV, respectively, as used by Keen et al.^52^ All of the other parameters were obtained by taking a linear interpolation between those of InAs and InSb. For GaInSb, the bowing parameter for the bandgap energy was set to 0.42 eV following Refaat et al.^53^ The remaining values were obtained through a linear interpolation between those of GaSb and InSb.

### 2.4 Transmission Electron Microscopy

A cross section of samples was prepared to investigate by TEM. Sample A was prepared as an electron-transparent lamella using a focused ion beam (FIB) in a Zeiss Auriga FIB-scanning electron microscope (SEM) dual-beam. In the case of Sample B, a traditional preparation method for semiconducting materials was used.^54^ After grinding, polishing, and dimpling, the cross section preparation of Sample B was finished by ion-milling in a vacuum chamber cooled with LN_2_ Gatan 691 Precision Ion Polishing System (PIPS). Eventually, both prepared samples were analyzed in a Thermo Scientific Talos F200X scanning transmission electron microscope (STEM) at an acceleration voltage of 200 kV.

## 3 Results and Discussion

### 3.1 Optical Characterization

**Figure 2:**
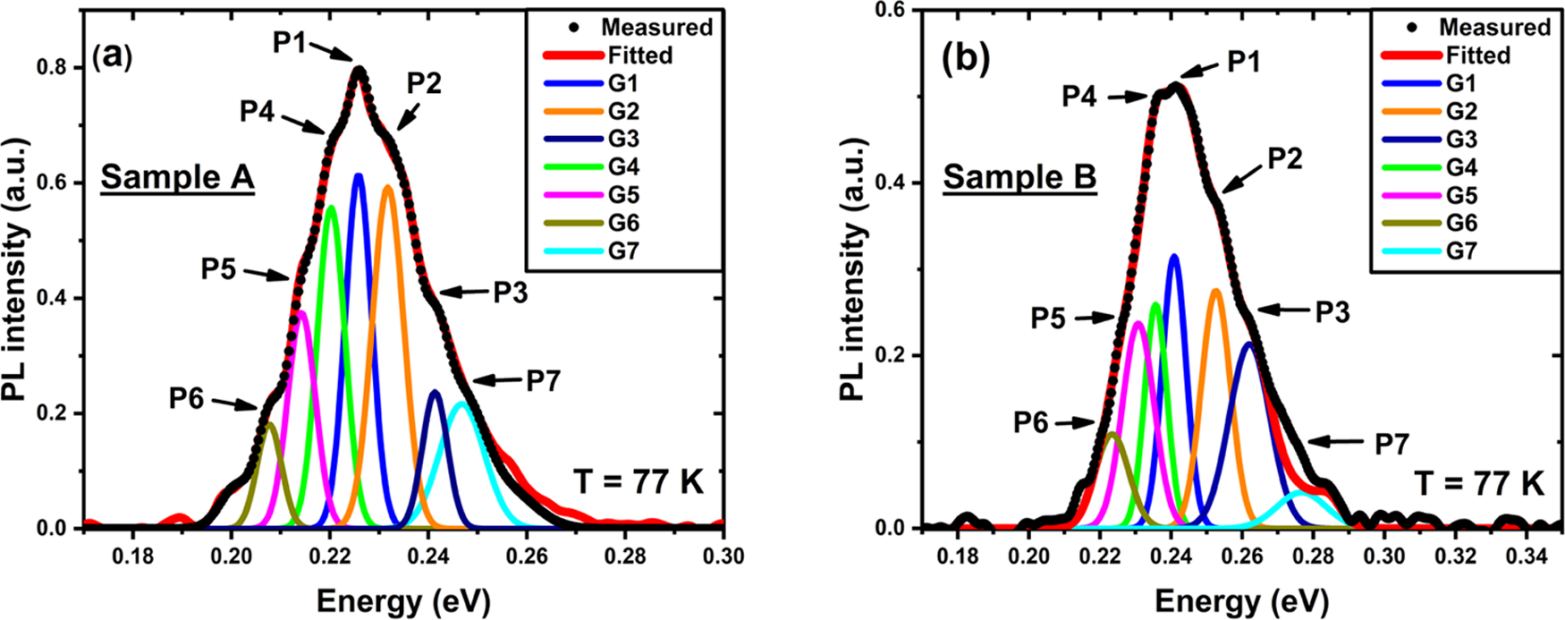
PL spectra at 77 K fitted with the Gaussians from P1 to P7 for (a) Sample A and (b) Sample B.

PL spectra at 77 K of Sample A (Figure 2a) and Sample B (Figure 2b) show shoulders (secondary peaks) around the intense primary emission peak. To understand the origin of these peaks, we fitted the PL spectra with seven Gaussian approximations labeled as G1, G2, G3, G4, G5, G6, and G7 using FITYK, an open-source peak fitting software.^55^ The intense primary peak (P1) and the other secondary peaks at higher energies (P2, P3, and P7) and lower energies (P4, P5, and P6) are all assigned. The Gaussians G5, G6, and G7 were fitted to ensure a good consistency between the measured PL spectra and the resulting envelope function. The parameters used to fit P1, P2, and P3 are summarized in Table 1. As can be seen from Figure 2a,b, the highest peak is P1, which corresponds to the first electron and heavy hole miniband transition (e_1_-hh_1_) with a full width at half-maximum (FWHM) of 27 and 25 meV for Samples A and B, respectively, which is consistent with reports on MWIR InAs/GaSb T2SLs.^56,57^ We can also see that the maximum PL intensity of P1 for Sample A is 1.6 times as high as that for Sample B.

**Table 1:** Summary of 77 K PL Peak Fitting Parameters (Peak Positions, Heights, and FWHMs) Used to Fit Gaussian Peaks of P1, P2, and P3

sample	peak	center (eV)	height (a.u.)	FWHM (eV)
A	P1	0.2258	0.614	0.0273
	P2	0.2317	0.592	0.0075
	P3	0.2414	0.289	0.0066
B	P1	0.2408	0.314	0.0254
	P2	0.2526	0.275	0.0096
	P3	0.2620	0.213	0.0134

It is possible that Sample A might have higher As compositions at the InSb IF layers. Since As has a high beam equivalent pressure (BEP), a considerable amount of As flux may be present at the growing surface even if the shutter remains closed.^58^ According to Kim et al.,^59^ the higher As compositions might bring about higher lattice disorder. Indeed, as measured by X-ray diffraction (XRD), Sample A has a significant compressive strain with a lattice mismatch with GaSb substrate, which is around +0.479% due to the overall thick InSb IF layers. At the same time, Sample B is nearly lattice-matched to the GaSb substrate.^46^ Consequently, the higher PL intensity of Sample A may not be attributed to the higher As compositions in the InSb IF layers. A possible reason for the higher peak in Sample A is that the T2SL sample grown with InSb IFs using the MEE method is known to have more substantial luminescence efficiency. This is because the cations can migrate longer distances in the MEE growth mode without anions. Therefore, 2-D layer growth mode can be more readily obtained.^60^ In addition, we observe a redshift of approximately 15 meV in P1 for Sample A compared to Sample B. The origin of the redshift behavior in P1 for Sample A can be attributed to the difference in the SL periodic thickness as measured by XRD,^46^ a similar feature was also commented in the past elsewhere,^43^ or atomic intermixing. This can significantly influence the PL peak energy and the corresponding wavelength. This will be discussed later in more detail using the band heterostructure simulations and TEM measurements.

The other secondary peaks (P2 and P3) are unrelated to the second heavy hole or split-off orbit bands since these transitions occur at much larger energy levels. Weisbuch et al.,^61^ suggested that the formation of one ML thick growth island can occur at the IFs of wells and barriers in the multi-quantum well (MQW) system, which affects the confinement energy and, therefore, the PL profile. This might be a plausible explanation for the origin of peaks P2 and P3. The presence of growth islands results in uncertainty in the well width, and thus the energy uncertainty for the system can be calculated using the following equation
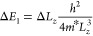
2where Δ*E*_1_ is the energy uncertainty, Δ*L_z_* is the well width uncertainty, *h* is Planck’s constant, m* is the effective mass, and *L_z_* is the well width. Therefore, considering the well width uncertainty to be 1/2 the lattice constant and the effective mass to be a weighted average of the effective masses of InAs and GaSb, the energy uncertainty is found to be approximately 7.2 meV. It is consistent with the variations observed for peaks 1, 2, and 3 (Table 1). On average, the energy difference between these peaks is marginally higher in Sample B than in Sample A, which is probably indicative of the energy uncertainty being slightly higher for this sample. This also agrees well with the measured XRD peaks, indicating that the overall periodic thickness, and therefore the well width, is slightly smaller for Sample B, which increases the energy uncertainty. Furthermore, this is consistent with the findings of Ashuach et al.^36^ and Kim et al.,^28^ who reported a high level of atomic intermixing and Sb segregation at the IFs of InAs/GaSb T2SLs, especially when an InSb IF layer is implemented.

The growth temperature is another key parameter that can easily facilitate the diffusion of atoms.^29^ As our SLs were grown at a temperature of 410 °C, distributions of In, Ga, and Sb atoms might occur, leading to the formation of quantum dot (QD)-like nanostructures. InGaSb QDs have been reported to emit at energies similar to peaks P4-P7 suggesting a presence of sub-ML QDs at the T2SL IFs.^62^ It has also been stated that the growth kinetics are favorable for the preferential formation of InGaSb QDs because the diffusion lengths are longer for the In atom than the Ga atom.

**Figure 3:**
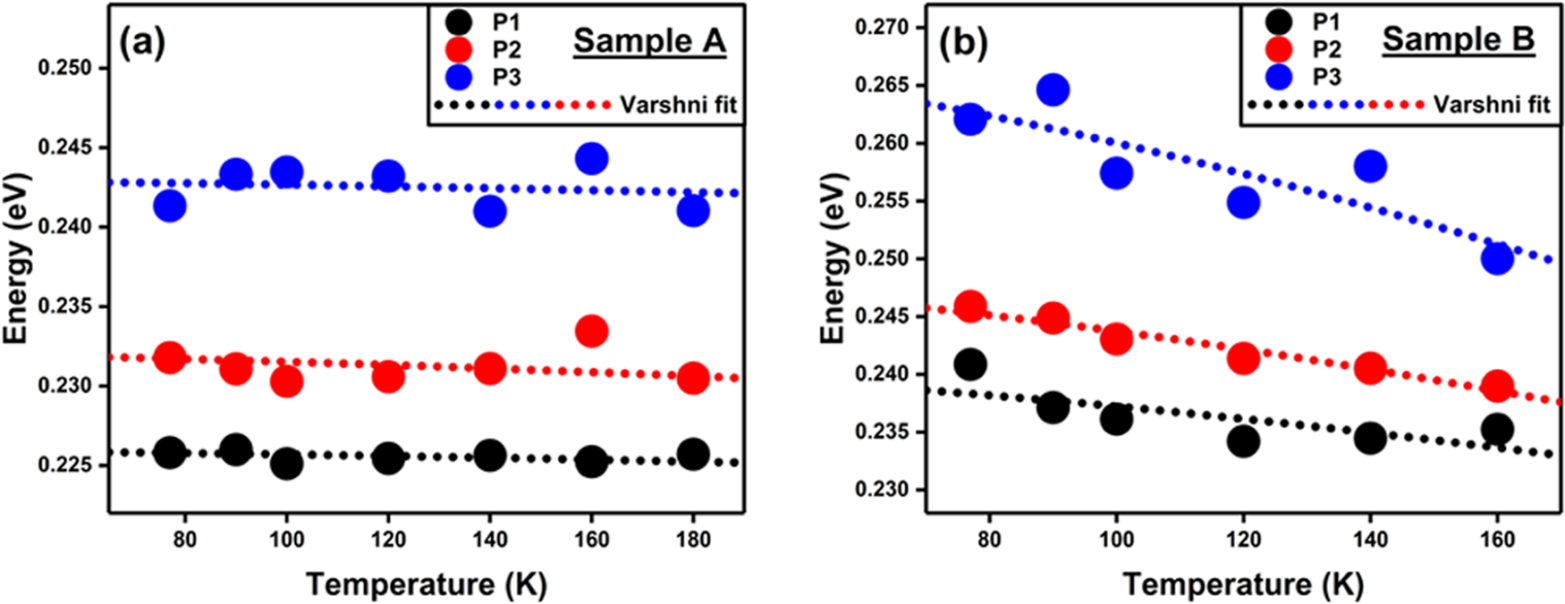
Bandgap energy versus temperature and fittings of peaks P1, P2, and P3 for (a) Sample A and (b) Sample B.

The bandgap energy versus temperature plots for peaks P1, P2, and P3 observed in Samples A and B are shown in Figure 3a,b, respectively. The temperature dependence of the bandgap energy was fitted using the Varshni equation^63^
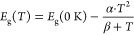
3where *E*_g_ (0 K) is the bandgap energy at 0 K, *T* is the temperature, and *α* and *β* are empirical fitting parameters related to the thermal expansion of the lattice parameter and Debye temperature, respectively. Here, *β* was fixed at 270 K based on a previous report.^64^ The *α* values for the prominent peaks P1, P2, and P3 were found to be slightly higher for Sample B than for Sample A. The *α* values and extracted bandgap energies at 0 K for P1, P2, and P3 for Samples A and B are included in Table 2. These *α* values are close to the other reported values in the literature.^65^ A smaller *α* value means that its bandgap is less sensitive to temperature variation, which implies that Sample A might be very suitable for high operating temperature (HOT) applications.^64^

**Table 2:** List of the *α* Values and Bandgap Energies at 0 K Extracted by Fittings of Peaks P1, P2, and P3 Using the Varshni Equation

sample	peak	*α*(eV/K)	*E*_g_ at 0 K (eV)
A	P1	1.000x10^-5^	0.225
	P2	2.000x10^-5^	0.232
	P3	1.053x10^-5^	0.242
B	P1	1.097x10^-4^	0.240
	P2	1.588x10^-4^	0.248
	P3	2.692x10^-4^	0.267

### 3.2 Band Heterostructure Simulation

As seen previously in Figure 2a,b, Sample A has a larger PL intensity than Sample B despite having a higher degree of structural inhomogeneity, as observed by the XRD measurement. This observation appears counterintuitive because the material inhomogeneity is expected to negatively impact the optical quality of a semiconductor via the increased presence of nonradiative defect centers. However, this corroborates the findings of Zhang et al.,^45^ who have noted that, when comparing T2SLs with InSb-like and mixed-like IFs, the InSb-like IFs were shown to give a stronger PL response despite having a greater structural inhomogeneity as measured by XRD. Considering that there is a high possibility of atomic intermixing and/or segregation as the InAs/GaSb heterostructure does not have common cations or anions, it is possible to explain this using band structure simulations of intermixing in a T2SL material system. Therefore, band heterostructure simulations were performed to further understand the influence of atomic intermixing and segregation at the T2SL IFs on the PL intensity.

**Figure 4:**
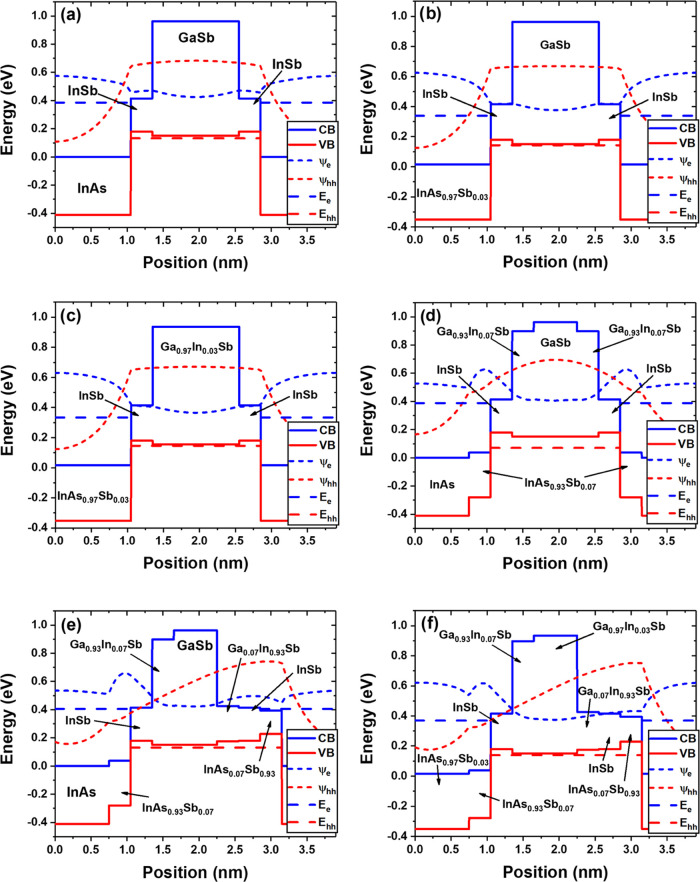
(a)–(f) Band structure and wave function overlap in the assumed scenarios S1–S6, respectively.

Herein, an 8-band k·p solver, implemented in the Nextnano3 software, was used to model the band structure of a 7 ML InAs/1 ML InSb/4 ML GaSb/1 ML InSb T2SL with various types of intermixing. One manifestation of the intermixing present in Sample A is that the binary InAs and GaSb layers appear to have small amounts of other elements, such as Sb or In. Furthermore, this phenomenon appears to happen more frequently at the IFs of the T2SL. Based on these speculations, we have simulated six scenarios (S1–S6) where we assumed various possibilities of IFs, illustrating the effect of various types of atomic intermixing on the optical properties of the T2SL. All of the simulations were performed at a temperature of 77 K. Having noted the above observations, we have assumed a T2SL with no intermixing 7 ML InAs/1 ML InSb/4 ML GaSb/1 ML InSb T2SL (S1) as one possible scenario. In addition, we propose other possible scenarios with an intermixing: 7 ML InAs_0.97_Sb_0.03_/1 ML InSb/4 ML GaSb/1 ML InSb T2SL (S2); 7 ML InAs_0.97_Sb_0.03_/1 ML InSb/4 ML Ga_0.97_In_0.03_Sb/1 ML InSb T2SL (S3); 5 ML InAs/1 ML InAs_0.93_Sb_0.07_/1 ML InSb/1 ML Ga_0.93_In_0.07_Sb/2 ML GaSb/1 ML Ga_0.93_In_0.07_Sb/1 ML InSb/1 ML InAs_0.93_Sb_0.07_ T2SL (S4); 5 ML InAs/1 ML InAs_0.93_Sb_0.07_/1 ML InSb/1 ML Ga_0.93_In_0.07_Sb/2 ML GaSb/1 ML Ga_0.07_In_0.93_Sb/1 ML InSb/1 ML InAs_0.07_Sb_0.93_ T2SL (S5); and 5 ML InAs_0.97_Sb_0.03_/1 ML InAs_0.93_Sb_0.07_/1 ML InSb/1 ML Ga_0.93_In_0.07_Sb/2 ML Ga_0.97_In_0.03_Sb/2 ML Ga_0.07_In_0.93_Sb/1 ML InSb/1 ML InAs_0.07_Sb_0.93_ T2SL (S6). The results of each one of these simulations are shown in Figure 4a–f, respectively, for scenarios S1–S6. The extracted parameters are given in Table 3.

**Table 3:** List of Parameters Extracted from Band Structure Simulations of Assumptions Made to Model Different Scenarios of Intermixing in S1–S6

scenario	intermixing	bandgap energy (eV)	wave function overlap (%)
S1	none	0.253	56.2
S2	Sb incorporation	0.191	52.2
S3	Sb and In incorporation	0.188	51.2
S4	symmetrical interfacial intermixing	0.318	65.6
S5	asymmetrical interfacial intermixing	0.275	62.4
S6	combination of S3 + S5	0.230	57.8

Figure 4b,c shows that both Sb incorporation in the InAs layer and In incorporation in the GaSb layer reduce the wave function overlap, which in turn reduces the optical response of the T2SL. However, Figure 4d,e shows that if the intermixing is confined to Sb or In incorporation at the InSb IF, both the electron and hole wave functions at the IF increase, increasing the overall wave function overlap. By comparing Figure 4a–f, it appears that the positive effect of the interfacial intermixing is more dominant than the negative effect of Sb or In incorporation, although, in reality, this will depend on the relative magnitudes of these phenomena. It can therefore be argued that intermixing will improve the optical response of most T2SL samples provided the introduction of nonradiative defect centers is not too severe (e.g., by the presence of a high density of lattice defects within the heterostructure).^66^Figure 4f presents a possible scenario in which both types of intermixing are present. This modeled scenario (S6) has a bandgap energy comparable to that of Sample A at 77 K but a larger wave function overlap than if its IFs were completely sharp, as shown in Figure 4a.

Comparing scenarios S1 and S6 in Table 3 also indicates that intermixing will likely reduce the bandgap energy of the T2SL, thereby increasing its cutoff wavelength. These simulation results corroborate the findings of PL measurements, as Sample A, the sample with greater intermixing, shows both a larger cutoff wavelength and a stronger PL peak. This also concludes that there is an urgent need for high-resolution transmission electron microscopy (HR-TEM) measurements to inspect the phenomena of intermixing at the T2SL IFs for both samples.

### 3.3 Structural Characterization

**Figure 5:**
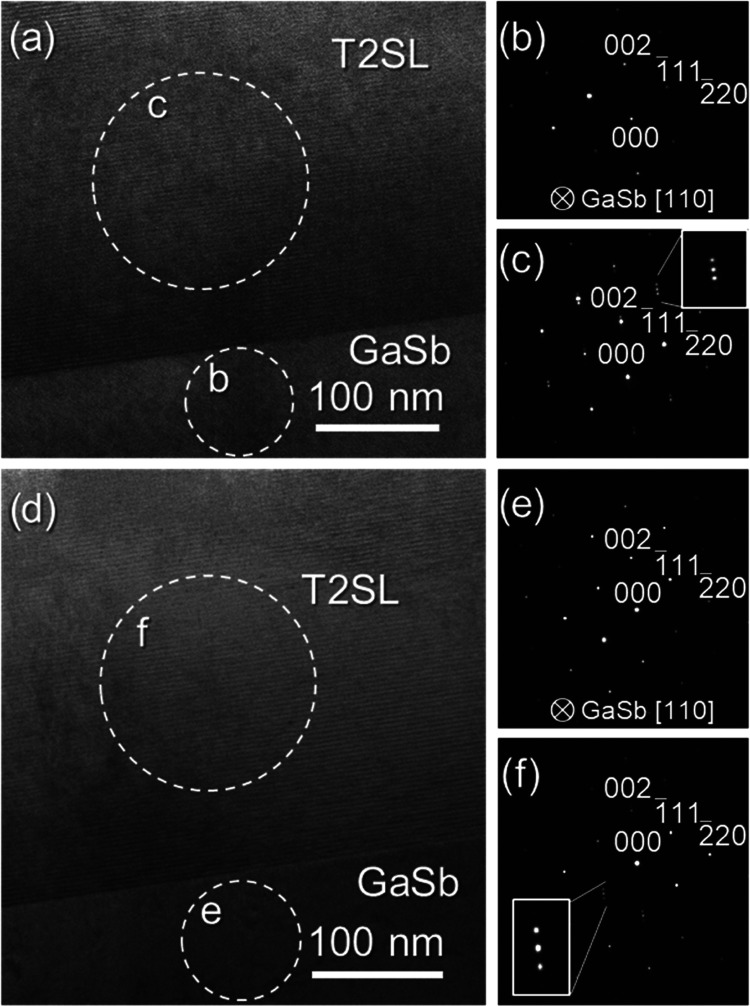
TEM micrographs taken along the [110] GaSb zone axis and SAED patterns from the GaSb buffer and the T2SL in Sample A (a–c) and Sample B (d–f).

Scanning transmission electron microscopy (STEM) technique is used in this work to study intermixing and atomic segregation phenomena in Samples A and B. Figure 5 compiles conventional TEM micrographs and selected area electron diffraction (SAED) patterns from both Samples A and B (Figure 5a–f, respectively). It provides a general overview of the crystallinity of these heterostructures at the microscopic scale. According to the TEM images in Figure 5a–d, it is confirmed that the T2SLs exhibit a homogeneous morphology in both samples. Also, there is a sharp transition from the GaSb buffer to the SLs, which will be confirmed later by compositional analysis. All of the micrographs and diffractograms depicted in Figure 5 were taken after tilting the TEM preparations so that one ⟨110⟩ zone axis of the GaSb buffer lies parallel to the observation direction. This can be checked in Figure 5b–e, which are SAED patterns taken around the GaSb region after reaching that zone axis. The observation of cubic materials along this direction is characterized by detecting a rhombus-shaped arrangement of reflections which (002) plane can be identified easily in one of the corners for both Samples A and B. It is worth remarking that the size of the circles included in Figure 5a–d, which indicate approximate regions where the patterns were registered, is not entirely representative of the actual size of the selected area apertures of the microscope that were employed (it always was the same size in fact). They are included only as a guide to indicate which material contributes to each pattern. In any case, two remarkable results can be extracted from these diffractograms. First, both SLs are single-crystalline since only ordered arrangements of bright reflections appear. Second, it is confirmed that they are epitaxially grown with respect to the buffer layer since their reflections are arranged in the same manner as those from GaSb when either material is observed under the same tilting conditions. Consequently, it is possible to identify the following epitaxial relations in both samples: T2SL [110]||GaSb [110] and T2SL [002]||GaSb [002]. Also, observing the patterns taken in the SLs more closely, some small reflections aligned in the growth direction with respect to the brightest ones can be identified. Two examples are marked and magnified in Figure 5c–f. These reflections are satellite spots, which, in the context of these systems, confirm the presence of long-period SLs. As a consequence, the components of the SL (e.g., InAs or GaSb) contribute to the diffractograms with their arrangements of reflections.

**Figure 6:**
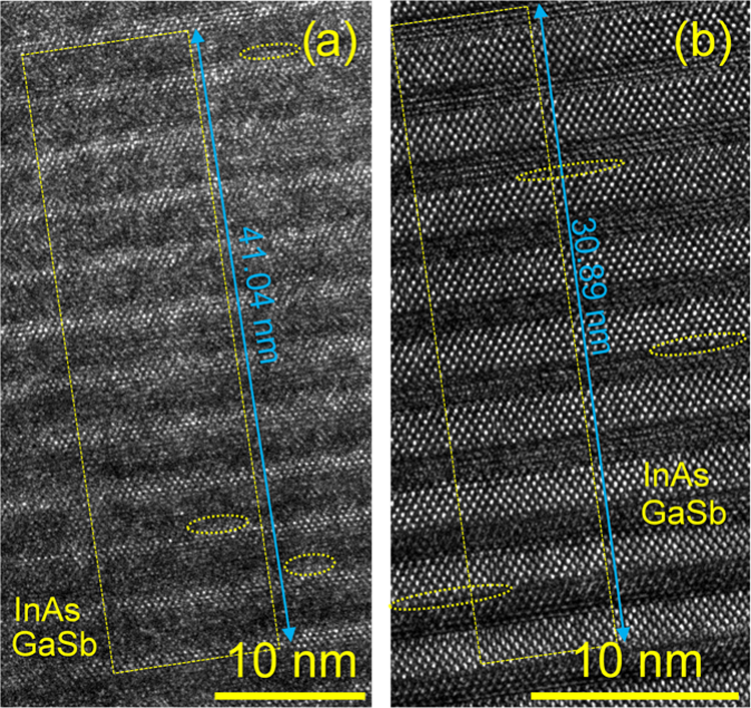
HR-TEM micrograph taken along the [110] GaSb zone axis from around the first bilayers of the T2SL in Sample A (a) and Sample B (b), marked with dotted circles regions with possible sub-ML QDs. The yellow rectangles indicate the region where the SL periods were averaged.

The SLs were subsequently inspected at higher magnification by means of HR-TEM. Figure 6a,b shows HR-TEM micrographs taken from the first layers of the T2SL in Samples A and B, respectively. At this magnification, some differences between the samples appear and are worth noting. First of all, although both InAs and GaSb can be identified as the main components in the micrographs of both samples (i.e., bright and dark films, respectively), the layers in Sample A look slightly rougher along its IFs than those in Sample B. This finding agrees with previous measurements of these samples by atomic force microscopy (AFM), which revealed that the T2SL without intentional InSb IF layers (Sample B) exhibited a slightly smoother surface than the SL with intentional InSb IF layers (Sample A).^46^ In addition, the thickness of each InAs/GaSb period is different in each sample. After measuring the GaSb buffer along the [002] direction as a reference, and assuming that this material is strain-free (*a*_0_ = 6.0959 Å),^67^ the lattice spacing for the (002) plane is calculated as 3.048 Å. In these images, the difference with this value along the same direction (measurements not shown) falls below 0.1 Å. Thus, it is possible to assume that the measurements of the period thickness are precise enough to reliably conclude that the T2SL in Sample A is generally thicker than that in Sample B. The values of the period thickness can be extracted from the distances given in both figures by dividing them by the number of complete SL periods that are contained within the yellow rectangles. These distances are determined using wide intensity profiles extracted from regions approximately delimited by the rectangles. The number of complete periods is equal to 11 and 9 for Figure 6a,b, respectively, and it is the highest possible in these micrographs without compromising the reliability of the intensity profiles and considering the low field of view already available in such local images. At the same time, the error made on averaging the SL period is still positively reduced by a factor of around 10 in both samples. This way, the resulting average period thickness values of Samples A and B after rounding are equal to 3.73 and 3.43 nm, respectively. This trend reasonably agrees with the period thickness values given elsewhere (3.69 and 3.54 nm for Samples A and B, respectively)^46^ and can be explained thanks to the intentional deposition of InSb at both IFs of Sample A. Consequently, the average thickness of each period increases in this sample with respect to the one expected in Sample B, which would be about 3.3 nm for a 7/4 InAs/GaSb T2SL (assuming that one ML would be about 0.3 nm thick). For the present study, these HR-TEM measurements reveal coherent thickness values, but for Sample A, they allow us to conclude that the InSb IF layers must be thicker than expected, a feature that was also commented on in the past.^46,68^ As a result, it seems that this excessively thick InSb IF layer led to a small roughening of the IFs in Sample A and, thus, a slightly lowered crystalline quality and increased strain.^46^ Controlling the thickness of InSb is important to obtain a lattice-matched InAs/GaSb SL since it is known that, although the progressively thicker InSb layers can help to reduce the lattice mismatch,^35^ they can lead to decreased structural properties and increased roughness if they start to become too thick,^43,57^ which appears to be the case in Sample A. In contrast, the transitions between films in Sample B are sharper and contain smoother IFs, as will be confirmed again shortly in the chemical characterization of these samples. However, it is reasonable to theorize that some of these regions of Sample A with increased roughness could be linked to the presence of subnanometer regions containing the previously mentioned sub-ML QDs. In Sample B, these hints are easier to find because of the deposited smoother layers, which allow the identification of a few short atomic rows at some random spots along the IFs, as indicated in Figure 6b. Somewhat similar features can be found in Figure 6a for Sample A, although it is harder to reach clear conclusions in this case because of the increased roughness of the SL, and we could be measuring InAs, InSb, or GaSb because of the resolution of the images available. In this sense, the comprehensive characterization of these sub-ML features is beyond the current stage of this research. Here, at least, we can identify some local thickness variations in both samples that could be related to the actual existence of such nanostructures. Nevertheless, considering the previous XRD analyses,^46^ one would expect that no significant changes in material quality are present in Sample A. In fact, those analyses revealed that the insertion of the InSb IF layers at both IFs indeed increased compressive strain. Still, the degradation of the material was not as significant after considering this result with other ones as a whole (e.g., obtained by PL spectroscopy or AFM) and compared to those from Sample B. Actually, the fast Fourier transforms (FFTs, not shown here) of the regions depicted in this figure demonstrate the validity of these observations since they allow us to observe the same typical behavior of a properly grown long-period SL that was already observed by SAED (Figure 5c–f), even though one could have expected in principle that, considering what can be observed by comparing the HR-TEM micrographs of Figure 6, Sample A would have a significantly more defective structure than Sample B. Consequently, in light of the above, it is possible to conclude that, from the perspective of structure and in agreement with the work mentioned above, the change in structural quality between these samples is not significant. Nevertheless, the addition of InSb, which is a material active in the infrared spectrum, still contributes toward a redshift in the optical activity of Sample A, which thus emits at a longer wavelength than Sample B. However, it appears that its thickness is still not high enough to motivate a significantly lowered crystallinity in Sample A, which was under a large compressive strain, while Sample B was found to be almost lattice-matched despite the absence of intentional InSb at both IFs.^46^ This can be explained by considering that the Sb-soak method selected for this specific system was adequate, since in other cases, the result of soaking with this element can also lead to a nonzero strain status,^69^ which can also happen if InSb is adequately deposited at both IFs.^38^ Consequently, we can conclude that the growth conditions in Sample A must be close to the optimum ones to obtain a lattice-matched InAs/InSb/GaSb/InSb T2SL with activity in the infrared spectrum, and this is reflected on the reduced crystallinity observed at the nanoscopic scale. Also, it is worth remarking that these findings agree with the observations previously given in this work. Namely, although Sample A exhibits an increased lattice disorder caused by features like a higher roughness, there are no lattice defects are present in a significant density throughout its structure, as Figure 5 proved. Therefore, as discussed in Figure 4, it appears that the improved optical response of Sample A can be explained by the existence of intermixing phenomena since there is an absence of an excessive density of nonradiative recombination centers.

**Figure 7:**
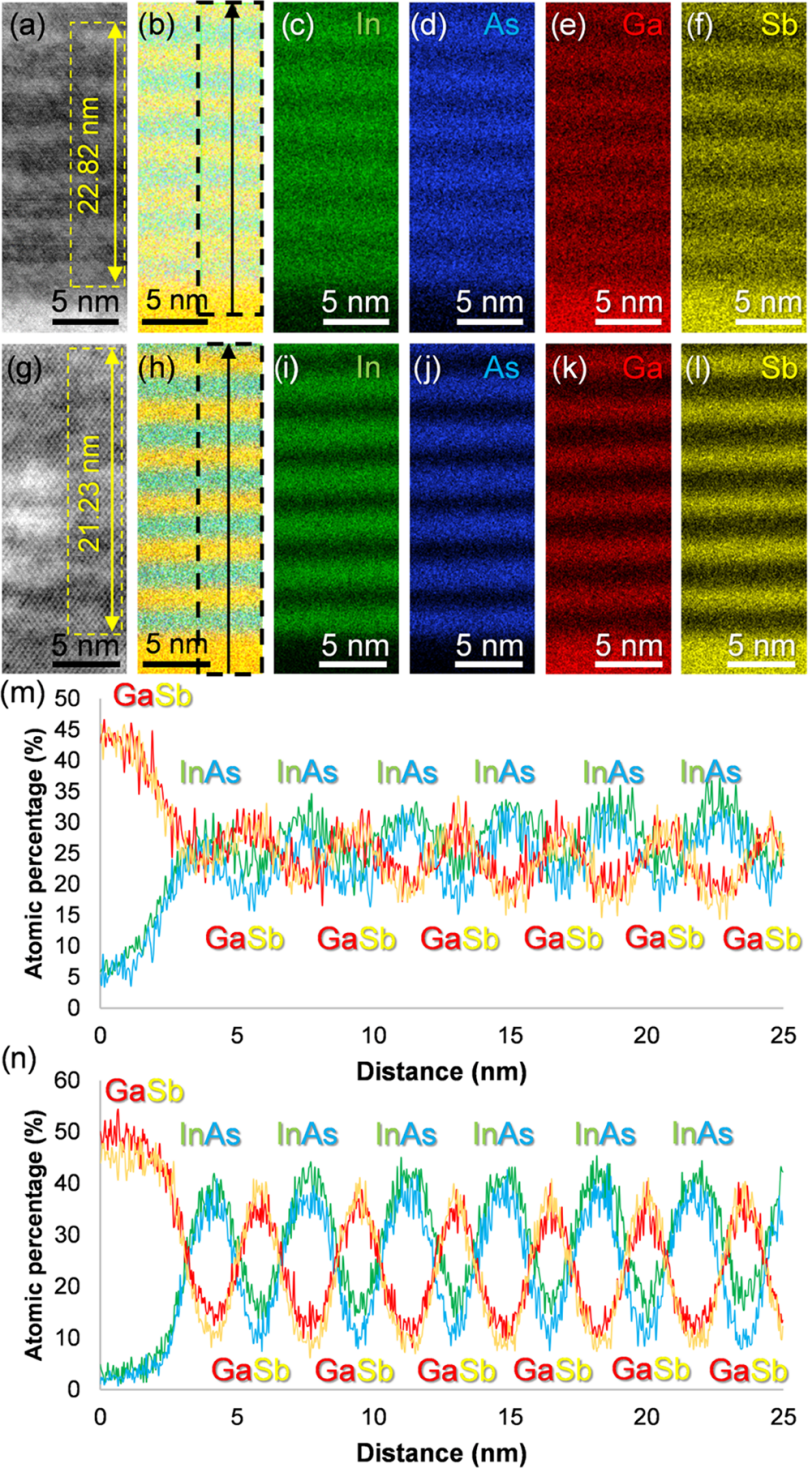
For Samples A and B, respectively: BF-STEM micrograph from the GaSb/T2SL region including areas with measurements of SL periods (a, g); combined In-As-Ga-Sb signals atomic percentage EDX map (b, h); individual maps by In (c, i), As (d, j), Ga (e, k), and Sb (f, l); and integrated atomic percentage profiles along the region marked in the combined signal maps (m, n).

The compositional analyses of these samples at this scale, including the further characterization of intermixing, were carried out by means of energy-dispersive X-ray spectroscopy (EDX). Figure 7 compiles the main results obtained after the characterization of both Samples A and B. This figure is structured as follows. First, bright-field scanning transmission electron microscopy (BF-STEM) taken at the T2SL/GaSb region of each sample is depicted in Figure 7a–g. These images allow us to distinguish atomic columns belonging to the different materials forming the heterostructures, and the interpretation of the contrast in the SLs is the same as in the HR-TEM images. The regions depicted in these micrographs were scanned by EDX to generate compositional maps, which are presented in Figure 7b–h for each sample. These maps contain the mixed signal of the four elements that were quantified: In (green, Figure 7c–i), As (blue, Figure 7d–j), Ga (red, Figure 7e–k), and Sb (yellow, Figure 7f–l). All of the maps indicate the atomic percentage of the represented elements. In the combined maps of Figure 7b–h, two rectangles with an arrow are included. Within these areas, the EDX mapping experiments were stable enough to reliably extract integrated atomic percentage profiles along the growth direction starting from the GaSb buffer. The results are shown in Figure 7m,n for Samples A and B, respectively. The integrated signal of all of the pixels in each slice of these rectangle-shaped areas is represented by one single point in each profile and gives a more accurate quantification of the regions under study. It is worth mentioning here that additional measurements of the periods of both SLs were carried out in Figure 7a,g to ensure the reliability of the results previously obtained by HR-TEM. In this case and using the EDX maps as guidelines to facilitate the measurements, up to 6 complete periods could be fitted within the yellow rectangles approximately inserted in each micrograph to obtain (following the same calculations as in Figure 6) average period rounded values of 3.80 and 3.54 nm for Samples A and B, respectively, which confirm the validity of previous experiments in this sense. Both sets of micrographs, maps, and profiles allow us to distinguish the sequences of stacked materials expected in each sample. However, the comparison between Sample A and Sample B in this context reveals differences in their compositional behavior. First, the four elements of interest look generally more scattered throughout the T2SL in Sample A since each map contains more diffuse signals than those in Sample B. In the case of indium and antimony, this feature is further supported by the presence of the intentional InSb IF layers in this sample, which would thus spread out In/Sb signals throughout the T2SL. As for gallium and arsenic (although this feature would also involve indium and antimony again), their apparently more scattered presence throughout Sample A can be explained by considering the slightly increased roughness of the bilayers in this sample. This feature could have led to material projections along the observation direction, meaning that contributions by any of these elements are more likely to happen at the IFs. The occurrence of this phenomenon could be possible by assuming that intensity signals for all of the elements in these regions decrease and increase more softly than in Sample B when the STEM probe reaches them and scans the next layer. Consequently, there would be apparently smaller compositional variations in the regions between consecutive films. However, taking into account the results shown up to this point (i.e., PL, HR-TEM), it is more reasonable to conclude that these presumably roughness-related projections are mainly related to the existence of compositional changes at the IFs (e.g., by intermixing phenomena). In any case, when the combined maps (i.e., Figure 7b–h) are compared again, it is possible to conclude that, besides this topic and considering both the presence of the InSb films and the mainly qualitative value of these EDX maps, both SLs are reasonably similar in terms of chemical composition. Although the IFs look sharper and the elemental distributions seem more homogeneous in Sample B, the similarity in the composition profiles of both heterostructures agrees with the other results reported in this work and contributes to explaining the differences between the samples. For example, the presence of InSb is confirmed in Sample A and explains the redshift in its optical activity and the increased degree of intermixing that can be observed along the IFs in the present STEM analyses. On the other hand, although Sample B contains sharper IFs and an SL with slightly higher structural quality than Sample A, it is worth remarking that one of the IFs (i.e., InAs-on-GaSb) was not controlled during its epitaxial growth.^46^ Consequently, intermixing phenomena at that IF is more likely to take place, although, as Figure 7 shows, it seems clear that such issues have taken place more frequently in Sample A. Even though the coverage of both IFs with InSb can take care of In/Sb segregation across the IFs if an adequate shutter sequencing is selected,^38^ our results agree more with those works where the implementation of InSb actually led to the increased occurrence of intermixing phenomena.^28,36^ In Sample B, considering that the IF as mentioned above is more prone to exhibit more extended intermixing phenomena than the GaSb-on-InAs IF according to other works^29^ (although it is worth commenting that the other IF can also be the most intermixed one),^30^ it is reasonable to assume that Sample B has been affected by this phenomenon at least in one IF, despite the fact that Sample A is generally more affected by these compositional heterogeneities. In any case, inspecting these IFs at higher magnification to check these hypotheses more thoroughly is the object of possible future experiments. Nevertheless, it is worth commenting that Figure 7m,n evidence the differences in growth methods followed for either sample. First, for Sample A (Figure 7m), it is possible to observe In/As and Ga/Sb peaks that are distributed in a reasonably symmetric manner along the growth direction with respect to their maxima and minima, which is expected considering the deposition process followed for this SL (i.e., GaSb/InSb/InAs/InSb). However, for Sample B (Figure 7n), the indium peaks are slightly displaced to the right compared to the arsenic peaks, so the GaSb-on-InAs IF contains an increased amount of indium which appears to agree with the Sb-for-As exchange growth technique selected for this SL. In any case, the lack of control in the growth of one IF of Sample B still implies that there is an increased chance of forming other interfacial materials between InAs and GaSb as a consequence of intermixing, which is an issue already reported and studied in InAs/GaSb SLs grown under different configurations and that can offer some advantages (e.g., modification of bandgap, as Sample A confirms).^26,29,34,70^ Since Sample B contains an almost lattice-matched SL, it is not expected that these intermixing phenomena took place in a significant extent throughout either IF of its heterostructure compared to Sample A, considering our previous findings. However, in light of the discussion provided up to this point, it is also reasonable to conclude that the potential apparition of this issue at localized spots of the SL cannot be fully disregarded. Consequently, this situation allows us to ultimately consider that both Samples A and B exhibit very similar qualities, but the former has the added advantage of exhibiting optical activity at a longer wavelength than the latter.

## 4 Conclusions

We have studied the effect of two different IF schemes on the optical and structural quality of the T2SLs. Sample A, with an intentional InSb IF layer, has shown a superior optical quality compared to Sample B, without an intentional InSb IF layer. It is observed that the maximum PL peak intensity is stronger in Sample A than in Sample B. The higher luminescence efficiency in Sample A could be attributed to the effect of the MEE growth mode. The cations can migrate longer distances without anions in the MEE growth mode. Hence, 2-D layer growth can be more readily obtained. We also studied the origin of different peaks in the T2SL samples and found that the prominent peaks are related to the SL transition. In contrast, other secondary peaks are associated with 2-D growth islands or sub-MLs of QDs at the IFs. Band heterostructure simulations were performed to speculate the observations made by PL measurements. It is concluded that the atomic intermixing and segregation not only change the bandgap energy of the T2SLs but also influence the wave function overlap and thus significantly affect the optical properties of the samples.

To verify the observations made by PL and band heterostructure simulations, TEM measurements were undertaken. It was shown that T2SLs exhibit a homogeneous morphology in both samples. Both SLs are single-crystalline since only ordered arrangements of bright reflections appear. It was also confirmed that the T2SLs are epitaxially grown with respect to the buffer layer. The insertion of the InSb IF layers at both IFs indeed resulted in an increase of compressive strain in Sample A. Still, the degradation of the material as a whole was not significant. It was also found that the IFs in Sample A are rougher compared to sharper IFs in Sample B, indicating an increased possibility of interfacial atomic intermixing and segregation. Therefore, according to these optical and structural characterization findings, it is believed that high material quality can be achieved by optimizing the growth on GaSb substrate by taking care of the IFs to enhance both the optical and structural properties of the T2SL heterostructures.

## Abbreviations

AFM: atomic force microscopy
APD: avalanche photodetector
BEP: beam equivalent pressure
BF-STEM: bright-field scanning transmission electron microscopy
EDX: energy-dispersive X-ray spectroscopy
FFT: fast Fourier transform
FIB: focused ion beam
FTIR: Fourier transform infrared
FWHM: full width at half-maximum
HOT: high operating temperature
HR-TEM: high-resolution transmission electron microscopy
IF: interface
LED: light-emitting diode
LN_2_: liquid nitrogen
MBE: molecular beam epitaxy
MCT: mercury cadmium telluride
MEE: migration-enhanced epitaxy
ML: monolayer
MQW: multi-quantum well
MWIR: mid-wavelength infrared
PIPS: precision ion polishing system
PL: photoluminescence
QD: quantum dot
SAED: selected area electron diffraction
T2SL: type-II superlattice
XRD: X-ray diffraction
